# Traumatic Triceps and Medial Ulnar Collateral Ligament Rupture in an Adolescent Mogul Skier: A Case Report

**DOI:** 10.7759/cureus.61026

**Published:** 2024-05-24

**Authors:** Ryan J Froom, YuChia Wang, Brandie Martin, Thomas R Hackett

**Affiliations:** 1 Orthopedic Surgery, The Steadman Clinic, Vail, USA; 2 Orthopedic Surgery, Steadman Philippon Research Institute, Vail, USA

**Keywords:** adolescent sports injuries, triceps rupture, snow-skiing, medial ulnar collateral injury (mucl), ulnar collateral ligament

## Abstract

Triceps tendon ruptures are uncommon injuries that account for less than 1% of all upper extremity tendon injuries. Medial ulnar collateral ligament injury (mUCL), while common in overhead athletes as a result of valgus forces during the throwing mechanics, has scarcely been reported in non-overhead, throwing individuals. Traumatic assault to the elbow may result in the rupture of the triceps tendon with concomitant mUCL injury. As such an injury pattern typically presents in middle-aged males, weightlifters, or American football players from eccentric overloading of the elbow. We present an adolescent, elite-level, competitive skier with traumatic onset distal triceps rupture with concomitant medial ulnar collateral ligament rupture suffered via a fall on an outstretched hand (FOOSH) mechanism. Magnetic resonance imaging (MRI) showed acute full-thickness avulsion of the distal triceps tendon occurring at the olecranon enthesis. An open tendon repair was performed, and the patient was able to report significant symptom resolution over the course of six months postoperatively and successfully return to elite-level competition. This was a unique and rare case of triceps tendon rupture with concomitant mUCL injury in an adolescent via a non-contact, high-velocity injury mechanism. While a rare injury combination, this case nevertheless identifies an area of research not currently extensively covered-trampoline training and associated injuries in adolescents. This case, therefore, not only adds a novel dimension to the understanding of triceps and mUCL injuries in young athletes but also underscores the need for heightened awareness and specific safety protocols in sports training involving equipment like trampolines.

## Introduction

Triceps tendon ruptures are uncommon, accounting for less than 1% of all upper extremity tendon injuries and 2% of all tendon avulsions. It is known to result in devastating outcomes unless appropriate and timely surgical intervention is performed [1,2]. Existing literature primarily concentrates on incidences in middle-aged men, weightlifters, and American football players, typically resulting from direct trauma or eccentric overload [3-6] and are rare in adolescents, with few case reports existing in the literature [7-11]. The two major mechanisms implicated for triceps rupture are falls on an outstretched hand (FOOSH) and direct injuries [12]. Triceps ruptures from a FOOSH mechanism can infrequently result in concomitant radial neck, capitellar, or medial ulnar collateral ligament (mUCL) injuries because of valgus load and remnant extensor mechanisms [12,13]. The medial ulnar collateral ligament (mUCL) is the primary restraint to valgus instability of the elbow [14]. It is commonly injured in overhead throwing athletes when a valgus force is focused onto the elbow [15-17]. In non-overhead throwing athletes, cases have been reported where the mUCL sustains damage via a traumatic insult [18].

We present a case exploring an unusual presentation of a complete triceps rupture and concomitant olecranon avulsion fracture as well as mUCL injury in a 14-year-old male, elite mogul skier, following a trampoline training incident. The rarity of such an injury in an adolescent athlete in the context of trampoline use through a FOOSH mechanism provides a unique presentation. The incidence of severe trampoline injuries is notably high, with a recorded annual incidence of 6.28/100,000 in children and adolescents [6,19]. Trampoline training, often utilized in sports for developing aerial skills, comes with its own risks, which are heightened in high-velocity sports such as mogul skiing.

## Case presentation

A 14-year-old male, elite-level mogul skier, presented to our institution with right elbow pain and edema following a fall onto an outstretched hand two weeks prior during trampoline training. Immediately after the fall, imaging studies obtained at a local emergency department revealed a traction fracture of the patient’s olecranon apophysis, and a complex tear of the triceps insertion on the ulna. No signs of dislocation or instability were reported. The patient reported immediate cessation of activity following the initial injury and presented to our institution for further evaluation.

On physical exam, mild swelling over the posterior aspect of the elbow and an abrasion on the posterior aspect of the elbow were notable. The patient’s olecranon and triceps tendon were tender to palpation but he did not report focal neurological deficits. His passive elbow extension range of motion (ROM) was 5 to 145°. The patient was unable to perform active range of motion testing or to extend the elbow against gravity, although full active and passive pronation and supination were observed.

**Figure 1:**
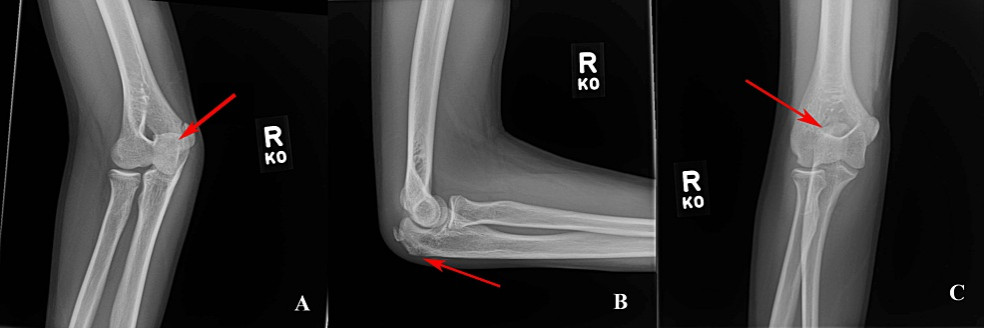
AP (A), lateral (B), and oblique (C) radiographs of the right elbow demonstrating an avulsion fracture of the olecranon. AP: anterior-posterior.

Initial elbow radiographs consisting of anterior-posterior (AP), lateral, and oblique were taken in the emergency room immediately after injury and demonstrated an avulsion fracture of the olecranon (Figures 1A-1C).

**Figure 2:**
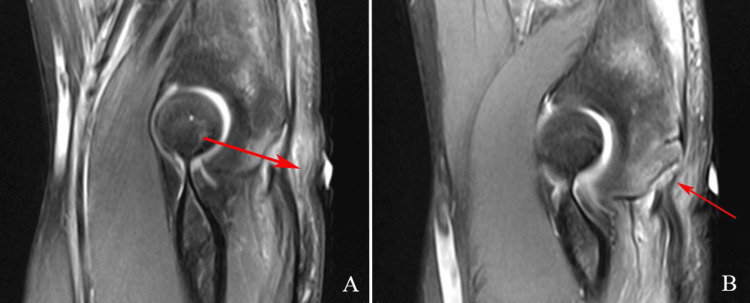
Magnetic resonance images (A) and (B) demonstrating acute full-thickness avulsion of the distal triceps tendon occurring at the olecranon enthesis.

Magnetic resonance imaging (MRI) revealed an acute full-thickness avulsion of the distal triceps tendon occurring at the olecranon enthesis with an associated 8-9 mm avulsion fracture fragment displaced along with the tendon (Figures 2A, 2B).

There was 12 mm of retraction of the torn triceps tendon fibers. Moderate strain of the flexor digitorum profundus muscle near the triceps injury and olecranon. The mild strain of the common flexor tendon at its origin without tear. Acute full-thickness avulsion/tear of the anterior band/bundle of the ulnar collateral ligament (UCL) occurring at the humeral enthesis with an associated 4-5 mm linear avulsion fracture of the olecranon attached to the torn fibers. There was a 3 to 4 mm of retraction of the torn UCL fibers, and a full-thickness tear of the posterior band/bundle of the UCL occurring at the humeral enthesis without retraction and bone bruising at the posterior aspects of the capitellum without fracture.

Surgical repair of the triceps tendon was indicated due to the extent of the injury as well as the patient's desire to return to mogul skiing, especially due to the triceps strength demand during the initial push-off as a high-level mogul skier. The decision was made to conservatively treat the UCL with immobilization since the patient was not a throwing athlete and would be immobilized at 60° for four weeks with no active elbow extension after the triceps repair.

The patient received preoperative intravenous antibiotics as well as a chlorhexidine scrub in the preoperative holding area, was then brought to the operating room, and was placed in the left lateral decubitus position. A curvilinear incision was created over the posterior elbow. Sharp, blunt, and electrocautery dissections were taken through the skin and soft tissues of the triceps fascia, which was split in line with its fibers. The triceps tendon was then identified. An avulsion fragment from the olecranon revealed a complete tear of the triceps tendon. This was mobilized, and the ends of the triceps tendon were debrided of any frayed disrupted tissue. The footprint on the olecranon was extensively debrided to allow for an appropriate surface for reattachment. Then, utilizing the standard technique, two 4.75-mm SwiveLocks were placed into the most proximal aspect of the olecranon.

Multiple FiberWire® sutures were then passed through-and-through the tendon, and this included sutures through up to the myotendinous junction with FiberTapes. Some of the sutures were subsequently tied, and the tails were then dunked into two additional anchors, which were placed more distally on the olecranon. It should be noted that very careful attention was paid to the preservation of the joint surfaces, and this included X-ray evaluation. The surgeon conducted an intraoperative interpretation to carefully evaluate the anchor placement position. The surgeon completed the SutureBridge-style transosseous-equivalent repair of the triceps after the X-ray confirmed the appropriate location.

The patient reported minimal pain medication utilization at one-week postoperative visit with no pain while adhering to physical therapy protocol with a targeted focus on starting elbow extension resistance exercises at eight weeks. At the seven-week postoperative visit, the patient had been attending physical therapy three times a week with an emphasis on range of motion progression. Twelve weeks postoperatively, the patient displayed improvement in his pain, range of motion (ROM), and strength. He was able to complete his stepwise progression for his return to sport by returning to competition six months postoperatively.

## Discussion

This case highlights the rarity of triceps tendon tears in the pediatric population, especially in non-contact sports athletes like mogul skiers. Our literature review revealed only four reported cases in skeletally immature patients, with diverse involvement ranging from distal to proximal triceps tendon ruptures and, in only one instance, an accompanying mUCL injury [9-12]. This case is particularly significant due to its occurrence following a trampoline accident, highlighting the unique injury mechanisms associated with high-velocity sports training.

Diagnosing triceps tears can be challenging in the acute phase, as they can be masked by excessive swelling. Clinicians should look for weakness in resisted extension and a palpable gap proximal to the olecranon [11]. While initial diagnostic imaging typically begins with X-ray, MRI plays a crucial role in confirming the extent of the injury [11]. Previous literature describes clinical decision-making guidelines stating if a patient can perform active elbow extension against gravity (manual muscle testing (MMT) >3/5), the injury is considered partial and may potentially be treated non-operatively with splint protection for four weeks at 30° flexion. If a tear of more than 50% is shown on MRI coupled with significant loss of triceps extension (MMT <3/5), then operative repair of the torn tendon is recommended [4]. The treatment approach varies based on the severity of the tear, with partial tears often managed non-operatively and complete tears in young athletes generally necessitating surgical repair [4,11]. Van Riet et al. recommend that, if surgical repair is to be performed, it should be done within three weeks of the rupture [3]. Notably, complete triceps tear repairs have shown positive outcomes, as evidenced in cases involving professional athletes [8].

The decision-making process in our case was influenced by several factors. These included the athlete's high level of activity, the demands of mogul skiing, which requires significant arm strength and mobility, and the nature of the injury itself. In discussing the potential risks associated with trampoline training, this case illuminates the balance between skill development and injury prevention. Trampolines, while effective for enhancing aerial skills and spatial awareness in sports like skiing and gymnastics, can expose athletes to unique injury mechanisms. The high-velocity and high-impact nature of these activities can lead to injuries that may not be commonly encountered in other training environments. This case, therefore, contributes to a growing body of evidence suggesting a need for increased vigilance and tailored safety protocols in trampoline training, particularly for young athletes.

In this particular case, the concomitant olecranon avulsion fracture, mUCL injury, and pediatric age required further consideration. mUCL injuries of the elbow are commonly found in overhead sports that impart a valgus load on the mUCL, such as baseball, volleyball, or javelin [20]. Many studies have reported on the outcomes of conservative treatment for UCL injury, primarily in overhead athletes, with satisfactory outcomes of athletes returning to their sports activities [20]. Our approach to this case included conservative treatment for the injured UCL and surgical intervention of the acute rupture of the distal triceps tendon and reincorporation of the olecranon avulsion fragment. This management allowed our patient to return to competitive mogul skiing after six months.

This case not only contributes to the limited literature on pediatric triceps tendon injuries but also highlights the importance of considering unique injury mechanisms in young athletes, particularly those involved in high-velocity incidents like trampoline training. It reinforces the need for vigilant safety protocols in training environments on trampolines in balancing skill development with injury prevention. More specifically, ensuring all athletes have introductory knowledge on trampoline use and proper falling technique. We also recommend training under the supervision of trained medical professionals, such as athletic trainers.

## Conclusions

This case study highlights the complex nature of trampoline-related injuries in young, high-velocity athletes and the challenges they pose in management. It emphasizes the importance of a thorough clinical evaluation and understanding of patient goals, especially in the context of pediatric sports medicine. The case advocates for a flexible and individualized approach to treatment, taking into account the unique demands of the athlete's sport and the potential for atypical injury presentations. It serves as a crucial reminder of the need for accurate diagnosis and specialized treatment strategies in managing sports-related injuries in the adolescent population. This case also underscores the importance of preventive measures and safety protocols in training environments, particularly when using equipment like trampolines, to mitigate the risk of severe injuries.

## References

[1] Inhofe PD, Moneim MS (1996). Late presentation of triceps rupture. A case report and review of the literature. Am J Orthop.

[2] van Riet RP, Morrey BF, Ho E, O'Driscoll SW (2003). Surgical treatment of distal triceps ruptures. J Bone Joint Surg Am.

[3] Farrar EL 3rd, Lippert FG 3rd (1981). Avulsion of the triceps tendon. Clin Orthop Relat Res.

[4] Kocialkowski C, Carter R, Peach C (2018). Triceps tendon rupture: repair and rehabilitation. Shoulder Elbow.

[5] Luthringer TA, Lowe DT, Egol KA (2021). Acute distal triceps tendon rupture repair: case presentation and surgical technique. J Orthop Trauma.

[6] Mair SD, Isbell WM, Gill TJ, Schlegel TF, Hawkins RJ (2004). Triceps tendon ruptures in professional football players. Am J Sports Med.

[7] Finstein JL, Cohen SB, Dodson CC, Ciccotti MG, Marchetto P, Pepe MD, Deluca PF (2015). Triceps tendon ruptures requiring surgical repair in National Football League players. Orthop J Sports Med.

[8] Kibuule LK, Fehringer EV (2007). Distal triceps tendon rupture and repair in an otherwise healthy pediatric patient: a case report and review of the literature. J Shoulder Elbow Surg.

[9] Sheps D, Black GB, Reed M, Davidson JM (1997). Rupture of the long head of the triceps muscle in a child: case report and review of the literature. J Trauma.

[10] Yeh PC, Dodds SD, Smart LR, Mazzocca AD, Sethi PM (2010). Distal triceps rupture. J Am Acad Orthop Surg.

[11] Zionts LE, Vachon LA (1997). Demonstration of avulsion of the triceps tendon in an adolescent by magnetic resonance imaging. Am J Orthop.

[12] Lee JH, Ahn KB, Kwon KR, Kim KC, Rhyou IH (2021). Differences in rupture patterns and associated lesions related to traumatic distal triceps tendon rupture between outstretched hand and direct injuries. Clin Orthop Relat Res.

[13] Khalil LS, Alkhelaifi K, Meta F, Lizzio VA, Shehab R, Makhni EC (2018). Complete rupture of the triceps tendon and ulnar collateral ligament of the elbow in a 13-year-old football player: a case report. J Orthop Case Rep.

[14] Alcid JG, Ahmad CS, Lee TQ (2004). Elbow anatomy and structural biomechanics. Clin Sports Med.

[15] Bruce JR, Andrews JR (2014). Ulnar collateral ligament injuries in the throwing athlete. J Am Acad Orthop Surg.

[16] Conway JE, Jobe FW, Glousman RE (1992). Medial instability of the elbow in throwing athletes. Treatment by repair or reconstruction of the ulnar collateral ligament. J Bone Joint Surg Am.

[17] Jones KJ, Osbahr DC, Schrumpf MA, Dines JS, Altchek DW (2012). Ulnar collateral ligament reconstruction in throwing athletes: a review of current concepts. AAOS exhibit selection. J Bone Joint Surg Am.

[18] Vaswani R, White A, Dines J (2022). Medial ulnar collateral ligament injuries in contact athletes. Curr Rev Musculoskelet Med.

[19] Runtz A, Nallet J, Font V, Anriot M, Pechin C, Langlais J, de Billy B (2022). Trampoline injuries in children: a prospective study. Orthop Traumatol Surg Res.

[20] Safran M, Ahmad CS, Elattrache NS (2005). Ulnar collateral ligament of the elbow. Arthroscopy.

